# Metabolic dysfunction associated fatty liver disease and coronavirus disease 2019: clinical relationship and current management

**DOI:** 10.1186/s12944-021-01564-z

**Published:** 2021-10-03

**Authors:** Yanlan Xu, Xinyu Yang, Hua Bian, Mingfeng Xia

**Affiliations:** 1 Department of Endocrinology, Zhongshan Hospital, Fudan University, Shanghai, 200032 China; 2 Department of Geriatrics, Qingpu Branch of Zhongshan Hospital, Fudan University, Shanghai, 201700 China; 3 Fudan Institute for Metabolic Diseases, Shanghai, 200032 China

**Keywords:** SARS-CoV-2, COVID-19, Fatty liver, MAFLD, Liver injury

## Abstract

The coronavirus disease 2019 (COVID-19) is caused by the severe acute respiratory syndrome coronavirus type 2 (SARS-CoV-2). At present, the COVID-19 has been prevalent worldwide for more than a year and caused more than four million deaths. Liver injury was frequently observed in patients with COVID-19. Recently, a new definition of metabolic dysfunction associated fatty liver disease (MAFLD) was proposed by a panel of international experts, and the relationship between MAFLD and COVID-19 has been actively investigated. Several previous studies indicated that the patients with MAFLD had a higher prevalence of COVID-19 and a tendency to develop severe type of respiratory infection, and others indicated that liver injury would be exacerbated in the patients with MAFLD once infected with COVID-19. The mechanism underlying the relationship between MAFLD and COVID-19 infection has not been thoroughly investigated, and recent studies indicated that multifactorial mechanisms, such as altered host angiotensin converting enzyme 2 (ACE2) receptor expression, direct viral attack, disruption of cholangiocyte function, systemic inflammatory reaction, drug-induced liver injury, hepatic ischemic and hypoxic injury, and MAFLD-related glucose and lipid metabolic disorders, might jointly contribute to both of the adverse hepatic and respiratory outcomes. In this review, we discussed the relationship between MAFLD and COVID-19 based on current available literature, and summarized the recommendations for clinical management of MAFLD patients during the pandemic of COVID-19.

## Highlights

• MAFLD was associated with increased risk and severity of COVID-19.

• 31% of COVID-19 patients had MAFLD.

• COVID-19 promoted liver injury and progression of MAFLD, especially in the youth.

• The mechanism of liver injury in patients with COVID-19 was multifactorial.

•Early screen and active treatment of MAFLD is recommended for patients with COVID-19.

## I**ntroduction**

COVID-19 is a new respiratory infectious disease caused by SARS-CoV-2 [1]. The World Health Organization (WHO) first informed of this new virus in Wuhan, China, on December 31, 2019 [1]. Since the beginning of the pandemic, more than 226.84 million cases and over 4.66 million deaths have been reported (as of September 17, 2021) [2]. Most infected patients had mild clinical symptoms, but about 15 and 5% became seriously and critically ill, respectively [1] The risk of severe COVID-19 is increased in the elderly population and those with past complications [3]. For the patients with COVID-19, the lungs are most frequently affected and the livers can also become an important target. Multiple studies have found that COVID-19 can cause liver injury, especially in the people with a previous history of liver diseases [4–6].

MAFLD is a type of liver disease associated with metabolic dysfunction. The diagnosis includes histological or imaging evidence of liver steatosis, plus one of the following three criteria, that is, type 2 diabetes mellitus (T2DM), overweight/obesity or metabolic disorders, regardless of alcohol consumption or other accompanying liver diseases [7]. The definition of MAFLD was recently proposed by a panel of international experts [8–10], which might better represent the hepatic manifestation of metabolic syndrome than the traditional definition of NAFLD. Compared with the definition of MAFLD, NAFLD overemphasizes the absence of alcohol use, but ignores the importance of metabolic disorders in the pathogenesis of liver steatosis. While the new diagnostic criteria of MAFLD provides a more accurate classification of the metabolic dysfunction-associated liver disease and eliminates the heterogeneity in the large range of patients with fatty liver. At present, the global prevalence of MAFLD was around 25%, [11] and MAFLD has become the most common cause of chronic liver disease (CLD) and the major causes of liver cirrhosis and even hepatocellular carcinoma in the world [7].

The studies on the relationship between MAFLD and COVID-19 might further deepen the understanding of the pathogenesis of COVID-19, and provide urgently needed clinical evidence for the treatment, management and prognosis of COVID-19 patients with MAFLD. As the epidemic continues, various degrees of liver injury have been observed in patients with COVID-19, and a large proportion of these patients suffered from both COVID-19 and MAFLD. However, for the patients with MAFLD, the risk of COVID-19 infection, the progression of both liver and respiratory disease, and the mechanism underlying the relationship between MAFLD and COVID-19 infection still have not been thoroughly investigated. Although emergency vaccination has been carried out in some countries, the inflection point for control of the epidemic has not yet appeared in the world. The current epidemic situation is still grim. This article will review the risks and challenges faced by MAFLD patients during the COVID-19 epidemic.

### MAFLD may be associated with the risk of COVID-19 infection

As an acute infectious disease, all populations are susceptible to COVID-19, regardless of race, sex, or age [12, 13]. However, some studies have found that patients with MAFLD seemed to have a higher proportion of COVID-19 than the general population. A recent meta-analysis included 6 studies with 1293 participants found that the comprehensive prevalence of MAFLD in COVID-19 patients was 31% [14], in comparison to the prevalence of 25% in the general population [11]. A report of 324 hospitalized COVID-19 patients in Shanghai diagnosed fatty liver using an insensitive X-ray computed tomography method, the serum aspartate transaminase levels were increased in the severe type of COVID-19 patients (34 U/L vs 23 U/L, *P* < 0.001), and the proportion of fatty liver was also marginally increased (34.6% vs 20.5%, *P* = 0.093) [15], which indicated the possibility of a correlation between MAFLD and COVID-19 infection. Therefore, it is speculated that MAFLD may be associated with the risk of COVID-19 infection, but it is still controversial whether the presence of MAFLD is a causal risk factor for the COVID-19 infection.

MAFLD is the liver manifestation of metabolic syndrome. A retrospective analysis based on extensive commercial databases in the United States found that the cumulative incidence of COVID-19 increased exponentially if metabolic syndrome existed (0.10% vs 0.01%) [16]. Among all co-morbid metabolic conditions, the presence of steatohepatitis, which was diagnosed based on electronic health records from nationwide healthcare systems from 1999 to 2019 in the United States, was most closely associated with COVID-19 [16]. These studies supported that patients with MAFLD should be regarded as a high-risk group for COVID-19, who might be susceptible to COVID-19 infection and its related complications.

In terms of mechanism, some studies have found that in the process of COVID-19 infection, SARS-CoV-2 first bind to the ACE2 receptors on the surface of host cells [17, 18]. In the study of animal model of hepatic steatosis, it was found that the increased expression of ACE2 could promote the entry of SARS-CoV-2 into hepatocytes and lead to liver injury [19]. In addition, social factors may also contribute to the susceptibility of MAFLD patients to COVID-19 infection. In the United States, low-income groups are more likely to suffer from metabolic diseases, such as obesity, diabetes or hypertension [16]. They are also more likely to be without health insurance, quarantined and fall into poverty. This group may be less concerned about health, which eventually leads to higher susceptibility to COVID-19. However, other studies have found that there is no difference in the expression of host genes required for SARS-CoV-2 infection between the non-NAFLD and NAFLD patients [20]. Consistently, animal studies have found that the expression of protein related to SARS-CoV-2 infection was not increased in the livers of MAFLD mice [20]. Thus, it seems impossible to use the increase of liver SARS-CoV-2 uptake to explain the increased COVID-19 infection in MAFLD patients. Moreover, some studies examined the effect of MAFLD-Genetic Risk Score on the risk of COVID-19, and found that MAFLD genetic risk was not associated to the risk of COVID-19 infection [21].

Taken together, there is a close clinical correlation between MAFLD and COVID-19 infection, but it is still controversial whether MAFLD was a causal factor related to the susceptibility of COVID-19, and the relevant mechanism underlying the clinical relationship between COVID-19 and MAFLD still requires further investigation.

### MAFLD increases the severity of COVID-19, but it may not alter the adverse outcomes

Patients with underlying diseases might lead to the poor prognosis of COVID-19. Metabolic diseases, such as hypertension, diabetes, obesity, and cardiovascular diseases, have been reported to be closely related to the adverse clinical outcomes the patients with COVID-19 infection [22–24]. Chronic liver disease also promotes the progression of COVID-19 [25, 26].

A retrospective study of 202 patients with COVID-19 found that the patients with progressive disease had a significantly higher percentage of NAFLD diagnosed by ultrasonography, and most NAFLD patients in this study could be diagnosed as MAFLD [27]. Multivariate regression analysis in this study showed that the presence of NAFLD was directly related with COVID-19 progression (OR 6.4[1.5–31.2]), manifested by longer viral shedding time and longer hospitalization days [27]. In another study of 110 patients with COVID-19 under 60 years old, the proportion of MAFLD increased from 43.7% in the non-severe COVID-19 group to 73.9% in the severe COVID-19 group (*P* = 0.01) [28]. After adjusting for age, sex, obesity, diabetes, hypertension and smoking status, the correlation between MAFLD and the occurrence of severe COVID-19 was significant (OR 4.07[1.20–13.79]) [28]. Another multicenter preliminary analysis of young and elderly patients also confirmed the relevance between MAFLD and severity of COVID-19 without considering sex, age, smoking and other accompanying metabolic disorders (OR 2.67[1.13–6.34]) [29]. As for the hospitalized COVID-19 patients, the history of NAFLD/nonalcoholic steatohepatitis (NASH), determined based on the electronic medical record data, was related to the increased admission rate of COVID-19 (OR 1.86; 95% CI, 1.43–2.42, *p* < 0.01) in a retrospective study of more than 6700 adults with positive SARS-CoV-2 RNA tests [30]. After adjustment for the history of NAFLD/NASH, the probability of hospitalization was significantly decreased in obese patients with COVID-19 [30], suggesting NAFLD/NASH as an obvious risk factor for COVID-19 related hospitalization [31, 32].

Therefore, the patients with MAFLD have an increased risk of developing severe type of COVID-19, with a longer virus shedding time, greater infectivity, higher hospitalization rate and longer hospitalization time.

The mechanisms of how MAFLD aggravates COVID-19 is still unclear. Patients with fatty liver are characterized by impaired hepatic innate immunity, for example, macrophages (M1 type) are in polarization stage, which will increase the levels of inflammatory mediators and cytokines and aggravate the COVID-19 infection [33, 34]. Among the profile of proinflammatory cytokines, interleukin-6 (IL-6) is a key component of cytokine storm [35]. In patients with fatty liver and obesity, the serum IL-6 level is positively associated with fat content in liver and viscera [36], and may promote the progression of COVID-19 [37]. In addition, patients with MAFLD are often accompanied by diabetes and obesity [38]. Diabetes has been proven to be associated to the adverse outcomes of COVID-19 [39, 40]. Hyperglycemia can damage the structure of the lungs, weaken the immune defense system, cause cytokine storm, promote lactic acid production, and change the inflammatory-immune response [39]. Similarly, obesity can also harm immune function and host defense mechanism [41]. Body fat accumulation makes the human immune system more susceptible to infection, and leads to lower response to antiviral and antimicrobial agents [42]. The other studies also found that the long-term elevated insulin levels in MAFLD patients were associated with reduced lung function [43], and the increase of leptin and reduction of adiponectin may mediate the deleterious effects of MAFLD on the airway inflammation and lung function.

MAFLD increases individual risk to develop severe type of COVID-19, but there was no significant difference in the rate of adverse outcomes, including intensive care unit (ICU) admission and mortality, between COVID-19 patients with and without MAFLD [31, 32, 44, 45]. A retrospective study of 193 patients with COVID-19 reported that after adjusting for confounding factors (male, age, hypertension, dyslipidemia and T2DM), the presence of fatty liver was not related with hospitalization in ICU (OR 1.14[0.53–2.5]) or hospitalization mortality (OR 0.86[0.44–1.69]) [31, 44]. Moreover, the Fibrosis-4 (FIB-4) score or the presence of liver cirrhosis was not significantly related to early clinical deterioration and adverse outcome of COVID-19 patients [44, 46–48]. In another retrospective study of 280 COVID-19 patients, no severe liver failure or liver-related complications was observed in the patients with fatty liver during hospitalization. It is considered that there is no significant difference in the disease complications and cilincal outcomes in COVID-19 patients with and without NAFLD [49].

Although many studies have shown that adverse outcomes are similar in COVID-19 patients with and without MAFLD, a meta-analysis found that patients with MALFD had an increased risk of ICU admission, with no significant difference in the overall mortality [31]. Moreover, a recent retrospective study also found that MAFLD with liver fibrosis was related to an increased risk of mortality and mechanical ventilation in COVID-19 patients [50]. Since the mortality is the most important final endpoint to assess the influence of MAFLD on the clinical prognosis of COVID-19, the current studies found inconsistent effect of MAFLD on mortality in COVID-19 patients, therefore, further large-scale prospective studies are still needed to clarify the effects of MAFLD on the mortality of COVID-19 patients.

In summary, most of current studies support the influence of MAFLD in the progression of COVID-19, but there is still no evidence that the presence of MAFLD will affect the prognosis of COVID-19.

### MAFLD-associated diabetes also promotes the progression of COVID-19

Diabetes and MAFLD are closely related with each other, and studies have demonstrated that about 70% of diabetic patients have fatty liver [51–53]. Diabetes has been fully recognized as an important risk factors of COVID-19 [39, 40, 54]. Diabetic patients with SARS-CoV-2 infection are more likely to develop severe type of COVID-19 [54–56]. Diabetes increased the risk of intubation, prolonged hospitalization days and increased mortality in patients with COVID-19 [57–59]. Good blood glucose can significantly reduce the mortality of COVID-19 patients [60]. It is also noticeable that although the frequency of blood glucose monitoring has been decreased in diabetic patients during the pandemic, their blood glucose control level was not affected [61]. Diabetes increased the levels of ACE2 receptors in the lung, oropharynx, tongue, and nasal airways, and hyperglycemia can induce abnormal glycosylation of ACE2 receptor and increase with the SARS-CoV-2 virus, which may increase the risk of SARS-CoV-2 infection in diabetic patients [62]. Elevated glucose levels can also increase the replication of SARS-CoV-2 directly [55]. These procedures may increase the infectivity and virulence of SARS-CoV-2 in diabetic patients [54]. In addition, hyperglycemia can directly damage lung structure, cause pulmonary dysfunction and aggravate lung injury [39, 54], and it can also affect the immune defense system, weaken the body’s ability to eliminate the pathogens, promote the infection of COVID-19 [39], and aggravate COVID-19 through the effect of cytokine storm [39, 54].

### COVID-19 infection promotes liver injury and disease progression in patients with MAFLD

Liver injury is common in patients with COVID-19 [4, 35, 63]. A retrospective study of 316 patients showed that the incidence of hepatic steatosis in the COVID-19 patients was 4.7 times higher than that in the negative control group (OR 4.698; 95% IC 2.12–10.41, *p* < 0.001) [64]. A retrospective study showed that among COVID-19 patients, 50% had liver injury on admission, and 75.2% had liver injury during hospitalization [27]. Most of the liver injury was mild and manifests as hepatocellular pattern, which was characterized by the increase of serum alanine aminotransferase (ALT), and about 33.2% patients showed persistent abnormal liver function during the hospitalization [27]. A study described the clinical characteristics of patients with concomitant fatty liver and COVID-19, and found that their serum ALT levels were significantly higher than those without COVID-19 infection [49]. Therefore, more severe liver injury was found in MAFLD patients with COVID-19 infection compared with those without COVID-19. Although liver injury may not be the main cause of increased mortality of COVID-19 patients, liver dysfunction will undoubtedly aggravate the patient’s clinical condition.

**Fig. 1:**
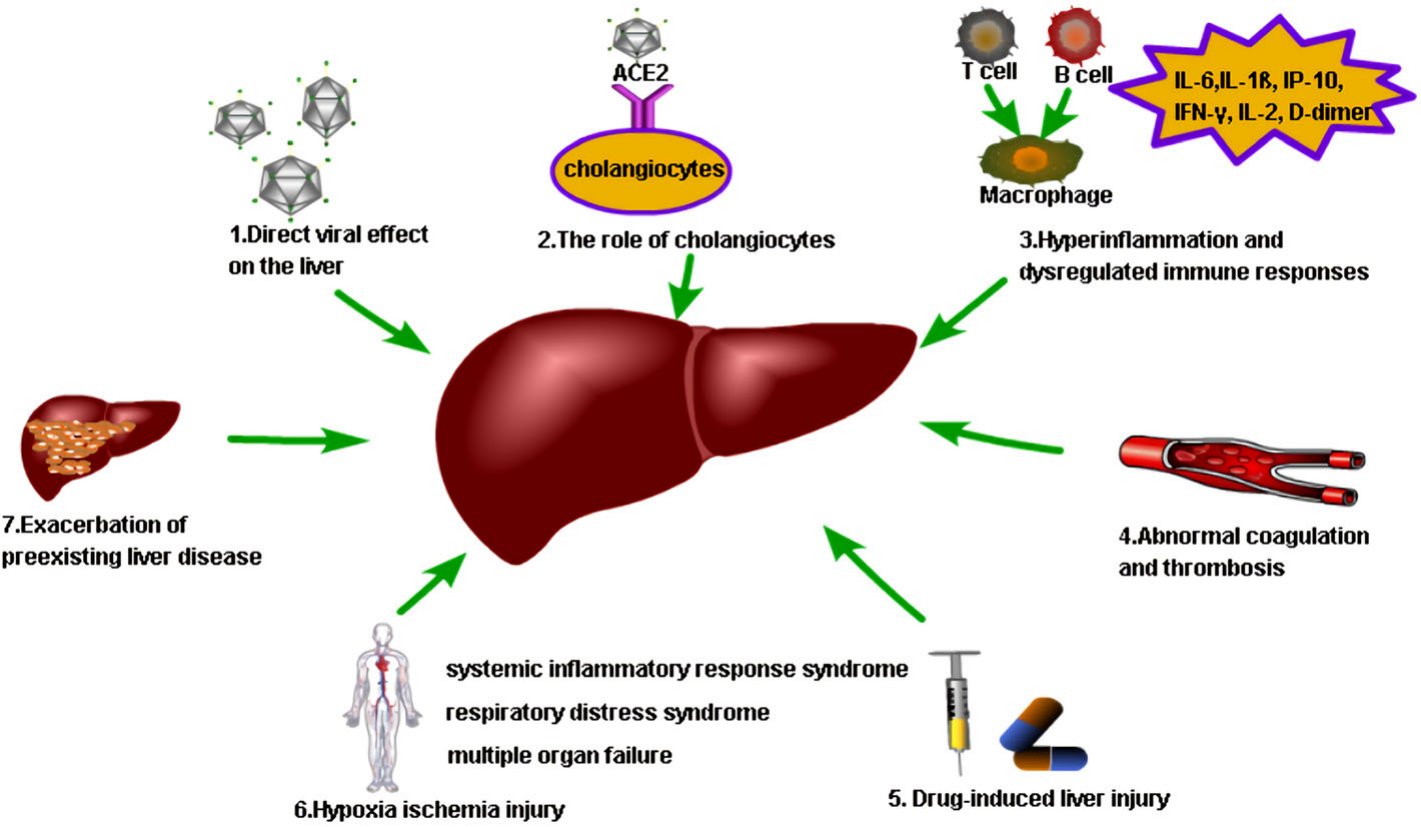
Possible mechanisms of COVID-19-associated liver injury in MAFLD patients, including the direct effect of the virus on hepatocytes, disruption of cholangiocyte function, whole-body hyperinflammation and dysregulated immune responses, abnormal coagulation and thrombosis, drug-induced liver injury, hepatic ischemia and hypoxia-reperfusion dysfunction, and the exacerbation of preexisting liver disease (including MAFLD)

The mechanism of COVID-19-related liver injury in patients with MAFLD is quite complex, involving a variety of factors. There are several speculations about the mechanism of liver injury related to COVID-19 infection, as shown in Fig. 1:

(1) Direct viral effect on the liver.

Studies have suggested that the liver injury may be directly caused by SARS-CoV-2 infection. SARS-CoV-2 in intestinal cavity can transfer to the liver through portal vein blood flow and enter the hepatocytes to cause liver injury through the ACE2 receptors [65]. A large number of coronavirus particles has been found in the cytoplasm of hepatocytes in COVID-19 patients [66]. Most virus particles had a complete coronal envelope, which indicates that SARS-CoV-2 can enter into hepatocytes and can further replicate in them [66]. Moreover, the ACE2 expression may be upregulated in hepatocytes after SARS-CoV-2 infection as a compensatory response, which might further enhance the deleterious effect of SARS-CoV-2 virus on hepatocytes [67, 68]. However, other studies have found that MAFLD is not related to the change of hepatic expression of COVID-19 infection-related genes, which does not support an increase of hepatic uptake of SARS-CoV-2 [20]. Thus, it is still controversial whether MAFLD will promote virulence of COVID-19 in hepatocytes.

(2) The role of cholangiocytes.

Angiotensin converting enzyme 2 (ACE2) is known to be the host cell receptor of SARS-CoV-2, which mediates SARS-CoV-2 infection [69, 70]. Some studies found higher expression of ACE2 in the cholangiocytes than the hepatocytes [65]. SARS-CoV-2 could also infect cholangiocytes directly, destroy their barrier and bile acid transporting function, cause bile duct dysfunction [71] and lead to hepatobiliary damage [5]. The biomarkers of cholangiocyte injury, such as alkaline phosphatase (ALP) and γ-glutamyltranspeptidase (GGT), were elevated in COVID-19 patients, suggesting a destruction of cholangiocytes in COVID-19 patients [5].

(3) Hyperinflammation and dysregulated immune responses.

Dysregulation of the innate immune response has been often observed in infectious patients, including COVID-19 patients [5, 72]. Some studies have reported an increase of neutrophil count and a reduction of T lymphocyte subsets such as CD3+, CD4+ and CD8+ T cell subsets in COVID-19 patients with liver injury [67, 73]. Patients with COVID-19, including mild and severe type of COVID-19, display elevation of inflammatory biomarkers and activation of T and B cell immune responses and macrophages recruitment [35]. Activated T cells and NK cells would secret a series of cytokines, including tumor necrosis factor alpha (TNF-α), interferon gamma (IFN-γ), and granulocyte-macrophage colony-stimulating factor (GM-CSF) [74]. Macrophages were also activated to produce a series of inflammatory factors [67, 68] such as IL-6, IFNγ, IL-2, IP-10, IL-1β, in patients with COVID-19 [75, 76]. Among them, IL-6 was found to play the most important role in “cytokine storm” in COVID-19 patients [35]. A significant increase in the level of IL-6 was also observed in patients with fatty liver [77, 78], which would activate the innate immune cell cluster in the liver [72] and drivers the progression of liver injury [79]. Monocyte chemotactic protein-1(MCP-1) (also known as C-C chemokine motif ligand 2, CCL-2) was also increased after SARS-CoV-2 infection [80, 81], which has been found to aggravate steatohepatitis [82] and promote the progression of MAFLD disease. In addition, overactivation of T cells was found in the liver histological biopsy of one patient with COVID-19, which showed the increase of Th17 cells and the high cytotoxicity of CD8 T cells, and contribute to the liver injury [83].

(4) Abnormal coagulation and thrombosis.

Abnormal coagulation and thrombosis are often found in patients with COVID-19. When infected with SARS-CoV-2, the virus may first infect endothelial cells and then cause diffuse endothelitis [84], which causes microvascular dysfunction and leads to hypercoagulability [85, 86]. The liver biopsies report of patients with COVID-19 showed massive dilatation of portal vein branches, lumen thrombosis, fibrin microthrombosis, hepatic sinusoid endodermatitis and hepatocyte necrosis [67, 87]. The COVID-19 patients with MAFLD had longer prothrombin time and higher levels of D-dimer, compared with their counterparts without MAFLD [88]. Therefore, abnormal coagulation and thrombosis may also correlate with liver injury in COVID-19 patients, and anticoagulant treatment might improve the disease prognosis [89].

(5) Drug-induced liver injury (DILI)

The treatment of COVID-19 usually incorporates different types of drugs, such as antiviral drugs, antibiotics, steroids, antipyretic drugs, etc. which have been recognized to be hepatotoxic [90]. The incidence of DILI is high in COVID-19 patients. A meta analysis showed that the pooled incidence of COVID-19 and DILI was 25.4% [91]. Widely used azithromycin, lopinavir, and interferon interferon have all been reported to cause liver cell damage or cholestasis [92, 93]. Antipyretic drugs (such as acetaminophen) are widely used in COVID-19 patients [94, 95]. Fatty liver can increase the hepatotoxicity of acetaminophen [96], and aggravate the original liver injury in MAFLD and may even cause the progression from fatty liver to steatohepatitis, or the aggravation of original steatosis, necrotizing inflammation, and liver fibrosis [90]. Usually, DILI was featured by moderate microvascular steatosis and mild lobular inflammation in the pathological liver examination of patients with COVID-19 [66]. However, none of the obvious pathological features of DILI, such as cholestasis, fibrin deposition, eosinophil infiltration, granuloma, massive central necrosis, or interface hepatitis, were found in liver pathology of COVID-19 patients [66] Thus, more research is needed to clarify its pathophysiology in patients with COVID-19.

(6) Hypoxia ischemia injury.

Severe complications were often found in patients with severe COVID-19, including systemic inflammatory response syndrome (SIRS), respiratory distress syndrome (RDS), and multiple organ failure (MOF), which can cause hypoxia and shock, result in hepatic ischemia, hypoxia and reperfusion dysfunction [35]. In critically ill patients, the peripheral and visceral blood flow would decrease, and eventually led to hypoxia of hepatocytes [97]. Then the hypoxia-inducible factors (HIFs), induced in the hepatocytes under hypoxia, could further exacerbate MAFLD [35, 98, 99].

(7) Exacerbation of preexisting liver disease.

Recent reports show that about 2–11% of COVID-19 patients suffer from potential chronic liver diseases (CLD) [100], such as fatty liver, viral hepatitis, and autoimmune liver disease. For patients with viral hepatitis who are receiving antiviral therapy, some drugs (such as biopharmaceuticals) may lead to virus activation during COVID-19 treatment, while stopping antiviral drugs or using glucocorticoids may also lead to viral hepatitis activation and liver injury [71]. Hypoxia, systemic inflammation, and circulatory disorder caused by COVID-19 can lead to secondary infection or decompensation of liver function in patients with previous liver disease [71]. Some studies have shown that elevated cytokine CCL-2 in COVID-19 may aggravate the progression from NAFLD to NASH and induce liver injury [101]. SARS-CoV-2 infection and its associated immune abnormalities are considered to be “multiple hits” to simple fatty liver, which may lead to liver injury and steatohepatitis [4].

To sum up, COVID-19 infection may increase the risk of liver disease progression in patients with MAFLD through various mechanisms. MAFLD and COVID-19 share a common inflammatory pathway [102]. This means that COVID-19 may accelerate the progress of MAFLD. The adverse hepatic outcome of the COVID-19 patients thus deserves extensive attention [103].

### Age influences the relationship between COVID-19 and MAFLD

A multicenter preliminary analysis of 327 patients showed that among severe COVID-19 patients, the proportion of MAFLD in young patients (< 60 years old) was significantly higher than that in elderly (> 60 years old). In severe type of young COVID-19 patients, the proportion of MAFLD was 55.9%, which was more than twice of that in the elderly patients (24%) [29]. It is noticeable that MAFLD was related to the severity of COVID-19 in the young but not elderly patients [29]. The mechanism of this age-related relationship is still unclear. Older patients have more comorbidities with multiple organs involvement, and higher mortality than younger patients, which may exceed the influence of MAFLD on COVID-19 [29].

### Recommendations for the management of MAFLD during the COVID-19 pandemic

The close correlation between COVID-19 and MAFLD have prompted us to strengthen the management of patients with MAFLD during the epidemic. Common suggestions for patients with MAFLD are similar to those for the general population, including perfect hand washing, social distance, strengthening personal protection, good manners for coughing, and avoiding sick people [25]. Lifestyle intervention (including weight loss suggestions, nutrition guidance, and diabetes management) may reduce the chance and severity of COVID-19 infection and slow down the progression of liver injury [84]. Considering the possibility of increase risk of severe COVID-19, early hospitalization is recommended for all MAFLD patients infected with COVID-19 [104]. It is suggested that patients with MAFLD and COVID-19 be given standard and timely diagnosis and treatment.

In addition, MAFLD patients may have other metabolic disorders, such as T2DM, obesity and hypertension, which may lead to increased mortality of COVID-19 patients [105, 106]. Monitoring and early management of these metabolic disturbance can minimize the risk of adverse prognosis in COVID-19 patients with MAFLD [106]. Although ACE2 is currently thought to mediate SARS-CoV-2 infection, there is still no conclusive evidence that angiotensin-converting enzyme inhibitors (ACEI) or angiotensin receptor blockers (ARB) can induce SARS-CoV-2 infection, or lead to aggravation of the disease, or even death from COVID-19 in patients with MAFLD [104]. It is still recommended to continue the treatment of hypertension following existing guidelines [104].

The specific recommendations for the management of liver injury in COVID-19 patients with MAFLD, include:

A) Early surveillance: Early accurate and repeated liver biochemical monitoring of COVID-19 patients can timely identify potential liver injury, and also help to reduce the risk of adverse drug events and achieve the best therapeutic concentration [25]. Although the best interval is unknown, it is recommended to monitor the changes of liver function tests of hospitalized patients with COVID-19 regularly [106], especially for areas with high prevalence of MAFLD. MAFLD-related tests should be carried out as early as possible.

B) Simplify treatment: Avoid repeated medication and pay attention to the dosage and duration of drugs, which may reduce drug-induced liver injury [71]. MAFLD patients may be susceptible to drug-induced liver injury [106]. COVID-19 patients, especially those with metabolic diseases such as obesity and diabetes, should be cautious in using drugs that may increase the risk of liver injury [90]. For patients with liver injury, suspicious drugs should be stopped in time when necessary.

c) Medication: For COVID-19 patients who have potential liver injury, taking antiinflammatory hepatoprotective drugs, such as ammonium glycyrrhizinate, may promote the recovery of the disease [35, 107]. L-ornithine-L-aspartate (LOLA) is also recommended for adjuvant therapy in patients with hyperammonemia and hepatic encephalopathy [71].

d) Supportive care: Hypoxic-ischemic injury can cause liver ischemia and hypoxia-reperfusion dysfunction [35]. Oxygen therapy is recommended for most hospitalized COVID-19 patients. For severe patients, it is recommended to timely improve pulmonary ventilation function and actively inhibit potential inflammatory storm [108], which will also inhibit the progress of liver disease and the aggravation of liver injury.

### Comparisons with other studies

Many previous studies have discussed the relationship between NAFLD and the progression of COVID-19, and this review focused on the relationship between the special type of metabolic dysfunction-related MAFLD and COVID-19 and incorporated many updated studies [14, 28, 37, 50, 90]. Different from most of the previous reviews with pure clinical studies, this review combines the current evidence from both clinical and animal studies and provides a comprehensive demonstration on the mechanism underlying the mutual effects between MAFLD and COVID-19 [19, 20]. In order to provide medical suggestions to COVID-19 patients with MAFLD, all the recommendations in this review have fully taken the liver condition of the patients into consideration, and might be more practical than the general COVID-19 suggestions for MAFLD patients [25, 35, 71, 90, 106–108].

### Strengths and limitations

Combined with a large number of literatures, this review comprehensively analyzed the relationship between MAFLD and COVID-19, especially the possible underlying mechanisms in detail. A practical suggestion for the management of COVID-19 patients with MAFLD was also provided in current review article. There are also several limitations in this review. First, there are not many articles on MAFLD and COVID-19, several studies on the correlationship between NAFLD and COVID-19 were cited in the current review. Second, liver biopsies were not performed in most studies, and the correlation between the liver histological features and COVID-19 could not be studied. Last but not the least, there are few studies on the correlation between COVID-19 and long-term progression of liver disease, and most of the included studies are retrospective studies, which did not permit an evaluation of the causal relationship between MAFLD and the risk of COVID-19 infection and progression.

## Conclusions and future perspectives

At present, the epidemic of COVID-19 is still continuing. Mounting evidence indicate that MAFLD patients will face greater risk of COVID-19 infection than the general population. With the increase of the global prevalence rate of MAFLD, a large part of this population may face serious risk of COVID-19. Although there are few reports of severe liver injury or liver failure directly caused by COVID-19, and liver injury does not seem to be the leading cause of death in COVID-19 patients, the presence of MAFLD and liver injury will undoubtedly worsen the clinical condition of patients as discussed in the review article.

For the patients with COVID-19, it is also recommended to screen for MAFLD and other chronic liver diseases in addition to general protection recommendations. Liver testing should be performed early for MAFLD patients with COVID-19 monitored during the disease treatment and long-term after recovery of COVID-19 to detect liver injury and liver disease progression in time. The therapeutic drugs for COVID-19 should be carefully selected, and those suspected to cause drug-induced liver injury should be carefully identified and discontinued if necessary. For critically ill patients, early prevention of inflammatory storms and respiratory support can also reduce the impact on the liver. At present, the launch of the vaccine may bring hope for alleviating the epidemic. However, MAFLD patients still face severe risks under the epidemic, so medical staff should carefully monitor and actively respond to the patients with MAFLD.

## Data Availability

All data generated or analysed during this study are included in this published article.

## Abbreviations

COVID-19: Coronavirus disease 2019
SARS-CoV-2: Severe acute respiratory syndrome coronavirus type 2
MAFLD: Metabolic dysfunction associated fatty liver disease
NAFLD: Non-alcoholic fatty liver disease
CLD: Chronic liver disease
NASH: Nonalcoholic steatohepatitis
ACE2: Angiotensin converting enzyme 2
T2DM: Type 2 diabetes mellitus
GRS: Genetic Risk Score
IL-6: Interleukin-6
ICU: Intensive care unit
ALT: Alanine aminotransferase
ALP: Alkaline phosphatase
GGT: γ -glutamyltranspeptidase
TEM: Transmission Electron Microscope
IFN-γ: Interferon gamma
GM-CSF: Granulocyte-macrophage colony-stimulating factor
TNF-α: Tumor necrosis factor alpha
MCP-1: Monocyte chemotactic protein 1
CCL-2: C-C chemokine motif ligand 2
DILI: Drug-induced liver injury
SIRS: Inflammatory response syndrome
RDS: Respiratory distress syndrome
MOF: Multiple organ failure
HIFs: Hypoxia-inducible factors
ACEI: Angiotensin-converting enzyme inhibitors
ARB: Angiotensin receptor blockers
LOLA: L-ornithine-L-aspartate
